# The Role of Artificial Intelligence in Enhancing Quality of Care in Nursing Homes: A Rapid Review

**DOI:** 10.3390/healthcare14111455

**Published:** 2026-05-25

**Authors:** Michael Mileski, Alejandra Mendoza Torres, Bradley Beauvais, Jose Betancourt, Zo Ramamonjiarivelo, Joseph Baar Topinka, Ramalingam Shanmugam, Roland Shapley, Rebecca McClay

**Affiliations:** 1 School of Health Administration, Texas State University, San Marcos, TX 78666, USA; a_m1719@txstate.edu (A.M.T.); bmb230@txstate.edu (B.B.); jose.a.betancourt@txstate.edu (J.B.); zhr3@txstate.edu (Z.R.); josephtopinka@txstate.edu (J.B.T.); shanmugam@txstate.edu (R.S.); rshapley@txstate.edu (R.S.); 2 School of Science, Technology, Engineering, and Math, American Public University System, Charles Town, WV 25414, USA; rebecca.mcclay@gmail.com

**Keywords:** artificial intelligence, nursing homes, skilled nursing facilities, long-term care, quality of care

## Abstract

Background/Objectives: The global aging population has placed escalating demands on long-term care systems, with nursing homes facing persistent challenges including chronic understaffing, high staff turnover, complex resident acuity, and elevated risk of adverse events. Artificial intelligence (AI)—encompassing machine learning, natural language processing, and computer vision—presents a transformative opportunity to address these systemic pressures by enabling proactive, data-driven care delivery. This rapid review aims to systematically map the existing literature on AI applications in nursing facilities, categorize how these technologies contribute to improvements in quality of care, and identify gaps warranting further investigation. Methods: Following Arksey and O’Malley’s framework and PRISMA-ScR guidelines, we conducted a comprehensive search of academic literature using a predefined Boolean string. The extracted data were organized and analyzed thematically. Results: The synthesized literature (n = 28 studies) revealed seven primary themes: (1) Clinical management, risk prediction, and monitoring; (2) Pressure injuries, wound management, and diagnostics; (3) Objective assessment, mental health, and end-of-life care; (4) Nutrition and personalized daily support; (5) Operational efficiency and staffing; (6) Technical, infrastructure, and economic barriers; and (7) Social, ethical, and demographic considerations. Conclusions: AI holds considerable promise for enhancing the quality of care in nursing homes across clinical, operational, and social domains. However, widespread adoption remains constrained by prohibitive infrastructure costs, data privacy regulations, algorithmic bias, staff resistance, and limited generalizability of findings across diverse populations. Successful integration requires evidence-based implementation frameworks and standardized and interoperable platforms.

## 1. Introduction

### 1.1. Rationale

The global population is aging at an unprecedented rate, placing immense pressure on long-term care systems [1]. Nursing facilities, also known as skilled nursing facilities (SNFs), are at the forefront of this demographic shift, caring for an increasingly frail and medically complex population. These facilities consistently grapple with systemic challenges, including chronic understaffing, high staff turnover, documentation burdens, and the need to manage multiple chronic conditions in residents [2]. These factors collectively threaten the ability of facilities to provide consistent, high-quality care, leading to increased risks of adverse events such as falls, medication errors, and hospital-acquired infections [3,4].

In response to these challenges, the healthcare sector has increasingly turned to technological innovation. Among the most promising of these are advancements in Artificial Intelligence (AI), which encompasses technologies such as machine learning, natural language processing, and computer vision. AI has the potential to fundamentally reshape care delivery by automating routine tasks, providing data-driven insights for clinical decision-making, and enabling proactive, personalized care models [5]. 

In the context of nursing facilities, AI applications are being explored to enhance resident safety, predict and prevent acute health events, alleviate administrative workloads, and combat social isolation [6,7]. For example, AI-powered sensor systems can passively monitor residents for fall risk or changes in mobility, while machine learning algorithms can analyze electronic health record (EHR) data to predict the onset of infections before symptoms become severe [7,8]. Despite this promise, the adoption of AI in long-term care settings lags behind that in acute care, and the evidence regarding its impact on quality of care remains fragmented. 

This rapid review maps the existing peer-reviewed literature on this topic. The primary objective is to identify the breadth and nature of AI applications currently being implemented or studied in nursing facilities, to categorize how these technologies aim to improve the quality of care, and to highlight the current gaps in knowledge that warrant further investigation. 

To date, most applications of AI in nursing homes have cast AI as a narrow, predictive tool. This paper proposes AI as an enabling infrastructure for continuous, data-driven improvement in providing quality care. This perspective differentiates our work from earlier studies and clarifies how AI can evolve from isolated tools to an integrative component of high-quality care delivery.

### 1.2. Objectives

To map the existing literature on AI applications in nursing facilities, categorize their impact on quality of care, and identify gaps warranting future research.

### 1.3. Population, Concept, and Context

Population: Residents of nursing homes/SNFs, including elderly adults with complex chronic conditions, dementia, and functional dependencies.

Concept: This rapid review focuses specifically on the benefits and drawbacks of the use of artificial intelligence in nursing facilities to improve quality of life.

Context: This rapid review primarily focuses on nursing homes.

### 1.4. State of the Current Literature

A growing body of research has begun to examine AI’s potential in long-term care settings, though the literature remains nascent and fragmented relative to acute care. Several prior reviews have mapped AI applications in geriatric and long-term care broadly. For example, Faiyazuddin et al. [5] and Fahim et al. [7] documented AI’s expanding role in diagnostics, treatment planning, and operational efficiency across healthcare sectors, but neither focused specifically on nursing home environments. Chustecki [6] offered a narrative overview of AI benefits and risks in healthcare, while acknowledging that evidence specific to long-term care settings is comparatively thin.

Within nursing home contexts specifically, earlier scoping reviews—including those by Zhao et al. [9]—have explored the concept of “smart nursing homes” and the acceptability of AI technologies among older adult populations, highlighting both enthusiasm for and skepticism toward these innovations. Work by Beltran et al. [10] examined clinical decision support systems in end-of-life care, and Fernandes et al. reviewed AI-based tools for detecting behavioral and psychological symptoms of dementia. Lee et al. [8] conducted a systematic review of digital healthcare for fall detection in older adults. Taken together, these prior studies suggest that AI in nursing homes has been studied primarily as a narrow, condition-specific tool—addressing falls, dementia, or wound care in isolation—rather than as an integrated infrastructure for continuous quality improvement.

Crucially, no prior rapid review has mapped the full breadth of AI applications across the major quality-of-care domains in nursing homes while simultaneously categorizing benefits, challenges, and evidence strength using an established appraisal model. This gap justifies the present review and shapes its primary contributions: a comprehensive thematic map, a comparative analysis of AI versus traditional care approaches across domains, and a consolidated agenda for future research.

### 1.5. Operationalization of Quality of Care

For the purpose of this review, “quality of care” is operationalized using the six-domain framework established by the Institute of Medicine (IOM) in Crossing the Quality Chasm [11] and widely adopted in subsequent academic and health services research: (1) Safety—avoiding harm to residents; (2) Effectiveness—providing evidence-based care and avoiding overuse or underuse; (3) Patient-centeredness—respecting residents’ values, preferences, and expressed needs; (4) Timeliness—reducing delays in care delivery; (5) Efficiency—avoiding waste of resources, equipment, and staff time; and (6) Equity—providing care that does not vary by personal characteristics such as race, ethnicity, or socioeconomic status. These domains served as an organizing lens for extracting and categorizing how AI applications in the included studies were purported to affect nursing home care quality.

## 2. Materials and Methods

### 2.1. Overview

The research process began with the researchers involved reviewing the manuscript’s intent and pertinent healthcare terminology. To guide the review, we applied PRISMA-ScR standards [12], the Kruse protocol [13], and Arksey and O’Malley’s framework for scoping studies [14]. A mix of these protocols was used for the rapid review due to the type of literature that was included. Four databases were queried to complete the literature review, including PubMed, CINAHL Ultimate, Academic Search Complete, and Nursing and Allied Health Reference Source. A broad three-concept Boolean search strategy was designed to maximize the sensitivity of retrieval, given the nascent and terminologically heterogeneous nature of the literature on AI in nursing homes. Initial piloting with narrower strings (e.g., restricting to ‘machine learning’ or ‘quality improvement’) excluded numerous relevant peer-reviewed articles; the broader strategy was therefore adopted to ensure comprehensive coverage. The exact Boolean string applied uniformly across all four databases was: (artificial intelligence OR AI) AND (quality OR improve OR enhance) AND (care OR life) AND (nursing home OR nursing facility OR SNF OR NF).

This string was entered into PubMed, CINAHL Ultimate, Academic Search Complete, and Nursing and Allied Health Reference Source using each platform’s standard advanced search interface, with no database-specific filters applied beyond the date range (1 January 2006–1 March 2026) and English-language restriction. The use of a uniform string across databases facilitates replication and is consistent with PRISMA-ScR transparency requirements.

### 2.2. Inclusion Criteria

Study search terms and Boolean operators were used to construct the database search string for article selection. The final Boolean string applied in each database was: (“artificial intelligence” OR “AI”) AND (“quality” OR “improve” OR “enhance”) AND (“care” OR “life”) AND (“nursing home” OR “nursing facility” OR “SNF” OR “NF”). This broader terminology was adopted after initial testing showed that narrower strings excluded relevant articles. The initial search, conducted between 18 February and 15 March 2026, yielded 309 articles published between 1 January 2006 and 1 March 2026. After screening, 28 peer-reviewed articles were deemed germane to the research questions and were retained for analysis; studies that fell outside the scope of the review or were duplicates were excluded. Any type of article published in a peer-reviewed journal (e.g., empirical studies, reviews, protocols) was eligible for inclusion.

### 2.3. Exclusion Criteria

**Figure 1:**
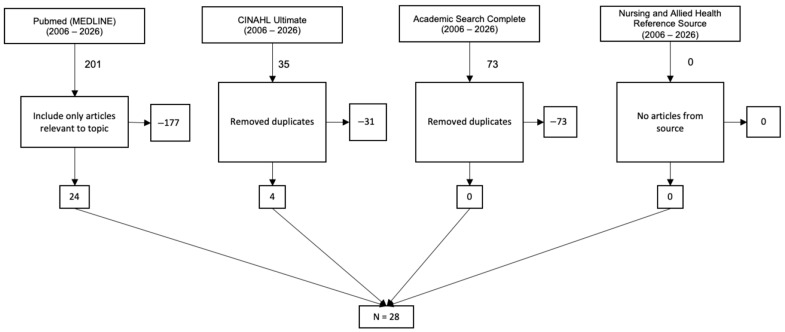
Preferred reporting items for rapid reviews and meta-analyses (PRISMA) diagram that demonstrates the study selection process.

Studies were included in the review only if they contained terms relevant to the Boolean search string and met predefined eligibility criteria. Non-English language publications were excluded. Only peer-reviewed journal articles were considered; gray literature, dissertations, reports, and conference abstracts were not assessed. Articles were retained only when all researchers agreed on their relevance. After applying these criteria, PubMed yielded 24 articles; CINAHL Ultimate and Academic Search Complete each contributed 4 articles, and Nursing and Allied Health Reference Source contributed none. All articles from Academic Search Complete were duplicates. This process resulted in a final sample of 28 unique articles. Figure 1 presents the PRISMA flow diagram for study selection.

The authors conducted a rigorous review of the 28 articles selected for inclusion in their analysis by reading the full manuscripts of each. At least three researchers agreed that any information extracted from individual articles would be included in the analysis. No discrepancies were reported among the researchers during the review of articles for inclusion or exclusion. In accordance with PRISMA-ScR reporting standards, a methodological quality appraisal was performed for all included studies using the Johns Hopkins Nursing Evidence-Based Practice Model (JHNEBP), which assigns evidence strength (Level I–III) and quality grades (A–C) to each article. Results are presented in Table 1. For the subset of empirical studies (quasi-experimental and non-experimental designs), additional appraisal was conducted using the Mixed Methods Appraisal Tool (MMAT) to assess risk of bias across domains, including sampling, measurement, and analytic rigor. Results presented in Table 2. Key limitations identified include reliance on single-facility samples, absence of control groups in several studies, and lack of blinding in observational designs. These methodological characteristics are acknowledged as limitations of the available evidence base (see Section 6) and should be considered when interpreting the thematic findings. Additionally, as some of the articles included were reviews, these do not typically appraise bias.

## 3. Results

### 3.1. Overview

Twenty-eight studies met the inclusion criteria for this rapid review, spanning publications from 2006 to 2025 and encompassing a mix of quasi-experimental, non-experimental, and qualitative designs. The majority of studies were conducted in high-income countries and focused on older adults residing in nursing homes or skilled nursing facilities. Using the Johns Hopkins Nursing Evidence-Based Practice Model, most articles were rated as level II (quasi-experimental) or level III (non-experimental or qualitative) with predominantly A or B quality ratings, reflecting moderate to high methodological rigor despite notable heterogeneity in design and outcomes. Collectively, the studies examined a diverse array of AI applications, which were organized into seven thematic domains: clinical management and risk prediction, pressure injury and wound diagnostics, objective assessment and mental health, nutrition and daily support, operational efficiency and staffing, technical and economic barriers, and social, ethical, and demographic considerations. The following sections summarize the key benefits and challenges identified within each thematic area.

Table 1 (above) presents the coding of the studies, along with their strength and quality, according to the Johns Hopkins Nursing Evidence-Based Practice Model (JHNEBP). While study design is generally an essential tenet of inclusion in this type of manuscript, it was not considered here due to the limited number of studies available for inclusion.

The quality results of the identified studies, as assessed by the JHNEBP methodology, demonstrate that the majority of the articles (57%) came from the level II (quasi-experimental studies) category. There were twelve level III category (43%, non-experimental or qualitative studies) articles used in the study. Given the limited volume of available literature, the research team included all relevant materials regardless of study design, while acknowledging variability in methodological rigor.

### 3.2. Thematic Findings

The analysis of the included literature revealed seven primary themes regarding the use of artificial intelligence to enhance the quality of care in nursing homes. Each theme included benefits and challenges, where applicable. To clarify how AI compares with traditional or non-AI approaches across key dimensions of quality, Table 3 summarizes the predominant direction of effect within each thematic domain.

#### 3.2.1. Theme 1: Clinical Management, Risk Prediction, and Monitoring

##### Benefits

Machine learning models, such as Bagging and Random Forest, have demonstrated superior performance over traditional scales by identifying critical risk factors, including fall history, age, mental status, and high-risk medications [15]. AI-enhanced Electronic Health Records (EHRs) are associated with a statistically significant 9% reduction in the rate of falls with major injury among long-stay residents [16]. Smart technologies integrate the Internet of Things (IoT) to enable real-time, continuous monitoring, allowing immediate detection of abnormal events [9]. Furthermore, remote surveillance can reduce fall-induced injuries and improve the efficiency of hourly rounding [17]. Clinical Decision Support (CDS) systems assist physicians in making prompt diagnoses by utilizing patient-specific data to alert them to “red flags” [10]. Predictive analytics can also proactively manage a resident’s care trajectory by predicting disease detection and mortality risk [10,18].

##### Challenges

Sensing technologies for activity recognition are often diversely applied and heterogeneous, making consistent clinical application difficult [19,20]. Additionally, there are valid concerns about the accuracy of AI predictions; for instance, some prognostic tools have shown poor discrimination in predicting mortality for specific conditions like advanced dementia [19].

#### 3.2.2. Theme 2: Pressure Injury, Wound Management, and Diagnostics

##### Benefits

The adoption of digital wound management solutions has led to an 11-fold reduction in high-level pressure injury citations (F686) and faster healing for sites like the sacrum and heels [21]. AI-driven mattresses automatically redistribute pressure based on real-time scans, improving sleep quality and reducing wound size [22]. Virtual wound care platforms also provided critical support during the COVID-19 pandemic [21]. Beyond wound care, AI-enhanced diagnostic tools for chest radiography allow non-radiology professionals to perform with accuracy similar to specialists, enabling earlier therapy initiation [23].

##### Challenges

Movement detection algorithms may record movements that do not result in the actual offloading of vulnerable tissue [24]. Furthermore, the microbial complexity of open wounds necessitates precise diagnostics to prevent ineffective treatment [25].

#### 3.2.3. Theme 3: Objective Assessment, Mental Health, and End-of-Life Care

##### Benefits

Digital measures provide objective bio-signals (e.g., heart rate variability) to help disambiguate pain from behavioral symptoms of dementia [26]. AI-enhanced EHRs contribute to a 59% reduction in residents with depressive symptoms and a decrease in antipsychotic use [16]. Technology also facilitates timely hospice and palliative care referrals and can trigger “goals-of-care” discussions, thereby reducing intensive care unit (ICU) transfers [10]. For social engagement, AI-driven voice companions significantly reduce depression and anxiety scores within four weeks [27]. Socially assistive robots and virtual caregivers stimulate engagement and facilitate digitalized cognitive assessments [28].

##### Challenges

Pain management must account for cultural and regional disparities, such as differences in analgesic use between Australian and Japanese facilities [29]. Additionally, there is a strong case against anthropomorphism, as human-like AI can lead to the “uncanny valley” effect, decrease trust, and undermine doctor-patient relationships [30].

#### 3.2.4. Theme 4: Nutrition and Personalized Daily Support

##### Benefits

Large Language Models (LLMs), such as Claude Opus and GPT, have achieved up to 94.2% agreement with human experts in classifying the healthfulness of food items [31]. These tools mitigate recall biases and identify residents at risk of malnutrition [31]. Machine learning can also predict personalized metabolic profiles based on gut microbiota 32]. In Smart Nursing Home (SNH) models, big data and AI provide services tailored to specific resident needs, supporting independence in activities of daily living (ADLs) [9].

##### Challenges

LLMs may exhibit a conservative classification bias, defaulting to “unhealthy” labels for ambiguous food descriptions. The accuracy of these models is also highly dependent on the dataset’s linguistic specificity [31].

#### 3.2.5. Theme 5: Operational Efficiency and Staffing

##### Benefits

AI-recommended models allow for individualized staffing that aligns with resident outcomes [33]. Analyzing care interactions has identified humor as a factor in “successful care,” which helps in improving caregiver training [34]. Smart technologies can also help address skilled caregiver shortages and automate routine family inquiries, reducing staff burden [35,36]. Furthermore, systems can pool information to suggest equitable treatment options and ensure proper resource allocation [10].

##### Challenges

Staff engagement with mobile documentation applications is often lower than expected [37]. Additionally, staff adherence to new AI training is frequently associated with the facility’s pre-existing quality rating [38]. Clinicians may also resist these tools due to fears of increased workload or a lack of trust in the technology [10].

#### 3.2.6. Theme 6: Technical, Infrastructure, and Economic Barriers

##### Benefits

Mapping and prediction tools help administrators identify geographical disparities in access to palliative and emergency care 39].

##### Challenges

The widespread adoption of IoT is hindered by prohibitive upfront costs and infrastructure limitations [20,36]. Data privacy regulations (such as GDPR) and the lack of standardized datasets limit the generalizability of AI research [38]. Continuous monitoring also raises significant concerns regarding privacy exposure. Furthermore, implementation complexity is high in low-income regions that lack stable internet and advanced IT infrastructure [10].

#### 3.2.7. Theme 7: Social, Ethical, and Demographic Considerations

##### Benefits

No benefits were found.

##### Challenges

Many older adults hold negative attitudes toward smart technologies, perceiving them as unnecessary or stressful to use [18]. If not carefully designed, AI systems can reinforce algorithmic bias related to socioeconomic, racial, or ethnic backgrounds [18]. Many tools also suffer from limited generalizability, as they are often validated only within specific patient subsets and may not translate across different cultural contexts [10]. Finally, technology adoption is often policy-driven rather than consumer-demand-driven, leading to a lack of genuine “smartness” in facility design [36].

## 4. Discussion

This rapid review synthesized evidence across seven thematic domains to examine how artificial intelligence can enhance the quality of care in nursing homes. The findings reveal a landscape of considerable promise tempered by persistent technical, ethical, and organizational barriers. Taken together, these themes underscore that while AI integration holds transformative potential for long-term care, its responsible implementation requires deliberate attention to equity, infrastructure, and interdisciplinary collaboration.

### 4.1. Clinical Impact and Risk Management

The most consistently supported domain was clinical management, risk prediction, and monitoring. Machine learning models demonstrated measurable improvements in fall risk stratification and injury prevention, with AI-enhanced EHRs associated with a statistically significant 9% reduction in fall-related major injuries. These findings align with broader literature indicating that AI-driven systems enable real-time monitoring of vital signs, earlier detection of deteriorating conditions, and more personalized care planning. The integration of IoT-enabled surveillance and Clinical Decision Support systems further extends the utility of AI beyond episodic assessment toward continuous, proactive care management. However, the heterogeneity of sensing technologies and documented inaccuracies in prognostic models for conditions such as advanced dementia indicate that clinical deployment must be accompanied by ongoing validation and context-specific calibration.

Compared with conventional approaches, the evidence indicates that AI-enabled risk prediction and monitoring generally outperform them on safety-related outcomes. Facilities adopting AI-enhanced EHRs and surveillance systems reported fewer major fall-related injuries and earlier detection of clinical deterioration than those relying on traditional fall scales, intermittent rounding, or retrospective record review, even though the effect sizes and study designs varied. In contrast, few studies directly compared AI with standard practice on resident-reported measures of safety or perceived security, leaving important dimensions of “high-quality” care under-evaluated.

### 4.2. Wound Care and Diagnostic Accuracy

Theme 2 highlights a compelling case for AI in wound management and diagnostics. The reported 11-fold reduction in high-level pressure injury citations and improved healing outcomes reflect the operational value of digital wound care platforms, particularly in resource-limited or pandemic-era settings. AI-enhanced diagnostic tools for chest radiography further expand the scope of non-specialist care, a meaningful development in settings where access to radiology is limited. Nevertheless, the challenge of movement-detection algorithms recording non-offloading movements cautions against overreliance on automated systems without nurse verification. The microbial complexity of chronic wounds similarly demands rigorous diagnostic precision that current AI tools may not uniformly provide.

### 4.3. Mental Health, Palliative Care, and Humanistic Considerations

The findings in Theme 3 are among the most nuanced of this review. AI-enhanced EHRs contributing to a 59% reduction in depressive symptoms and decreased antipsychotic use represent a clinically significant outcome in a population historically prone to overmedication. AI-driven voice companions and socially assistive robots demonstrated measurable reductions in depression and anxiety scores, supporting their utility for social engagement in isolated residents. Yet the challenge of the “uncanny valley” effect—wherein overly human-like AI undermines resident trust—points to an important design principle: AI in emotional and palliative care contexts should augment, not simulate, human connection. Cultural disparities in pain management further illustrate that AI tools cannot be assumed to generalize across demographically diverse populations without targeted validation.

### 4.4. Nutrition, Staffing, and Operational Efficiency

Large Language Models achieved up to 94.2% agreement with clinical experts in food classification, suggesting meaningful potential for nutritional surveillance and malnutrition risk identification. In staffing and operations, AI-recommended models showed the capacity to individualize workload distribution and reduce administrative burden on caregivers. These findings are particularly relevant given the global skilled nursing shortage, which demands innovative approaches to workforce sustainability. However, the documented gap between anticipated and actual staff engagement with mobile documentation tools highlights that technological capability alone is insufficient. As the literature consistently suggests, successful AI adoption is shaped as much by organizational culture and trust as by technical functionality.

Across Themes 2–5, AI-enabled tools tended to confer the clearest comparative benefits in domains that depend on rapid information processing and standardized responses—such as pressure-injury prevention, diagnostic accuracy, nutritional risk stratification, and workload optimization. Compared with usual care, digital wound platforms, AI mattresses, and virtual consultations were associated with fewer serious pressure-injury citations and faster time to appropriate treatment, while AI-based dietary classification and staffing algorithms showed promise for targeting resources to residents and units with the greatest needs. However, improvements in overall resident experience, therapeutic relationships, and staff well-being were more variable, underscoring that AI can strengthen the technical quality of care without automatically enhancing its humanistic or relational components.

### 4.5. Structural and Ethical Barriers to Implementation

Themes 6 and 7 collectively surface a set of structural and ethical challenges that transcend any single clinical application. The prohibitive upfront costs of IoT infrastructure, compounded by data privacy regulations such as GDPR and the absence of standardized datasets, create systemic inequities in AI access—disproportionately affecting low-income and rural facilities. These barriers risk widening existing disparities in long-term care quality rather than ameliorating them. Algorithmic bias represents a parallel concern; when AI systems are trained on non-representative data, they can systematically underestimate the care needs of minority or socioeconomically disadvantaged residents. The finding that technology adoption is frequently policy-driven rather than demand-driven further underscores the risk of implementing “smart” infrastructure that is nominal rather than functionally transformative.

From a transferability standpoint, the studies included in Themes 6 and 7 highlight that findings from high-income, technologically advanced facilities—predominantly in North America, Northern Europe, and East Asia—cannot be assumed to generalize to low-resource or culturally distinct settings. The conditions enabling AI success (stable broadband infrastructure, standardized EHR platforms, regulatory frameworks for data use) are precisely the conditions absent in the facilities most at risk of care quality deficits. This creates a structural paradox: the facilities with the greatest potential to benefit from AI are the least equipped to adopt it. Future research must explicitly address this transferability gap by conducting implementation studies in under-resourced facilities and diverse national contexts.

On ethical grounds, the absence of resident and direct-care worker co-design in the majority of included studies represents a significant gap. Algorithmic systems that affect care delivery—from staffing allocation to fall risk scores—embed value judgments that should be subject to democratic and clinical scrutiny, not delegated solely to developers or administrators. The principle of beneficence demands not only that AI does no harm, but that it actively promotes resident well-being as defined by residents themselves. Future governance frameworks must mandate bias auditing, transparency in algorithmic decision-making, and meaningful resident representation in AI evaluation processes.

### 4.6. Implications for Practice and Policy

These findings carry direct implications for nursing home administrators, policymakers, and clinical informaticists. First, AI tools should be integrated within evidence-based implementation frameworks that include staff training, change management protocols, and iterative performance evaluation. Second, procurement decisions should prioritize interoperable, standardized platforms that reduce fragmentation across clinical domains. Third, regulatory bodies should develop AI-specific governance frameworks for long-term care that address data sovereignty, liability for algorithmic error, and mandatory bias auditing. Finally, resident and family engagement in AI co-design is essential to ensure that deployed technologies reflect the values and preferences of those they serve.

### 4.7. Comparative Implications for High-Quality Care

Taken together, the comparative patterns across themes suggest that AI currently adds the most value to quality of care in nursing homes by augmenting safety, effectiveness, and efficiency. AI-enabled systems more reliably detect early signs of clinical deterioration, reduce high-severity pressure injuries, and streamline documentation and staffing processes when compared with traditional manual workflows and non-AI digital tools. At the same time, evidence that AI improves resident-reported outcomes, therapeutic relationships, and staff well-being relative to usual care remains limited and mixed, particularly in socially and demographically diverse settings. From a quality-of-care perspective, these findings imply that AI is best conceptualized as a complement to—rather than a replacement for—human and organizational investments in person-centered, equitable care. Future research should prioritize head-to-head evaluations of AI and non-AI care models using standardized quality metrics that capture both technical performance and experiential outcomes.

## 5. Conclusions and Future Directions

This rapid review demonstrates that artificial intelligence is already contributing to measurable gains in specific dimensions of nursing home quality, particularly safety, effectiveness, and efficiency of care processes. At the same time, the evidence base for AI’s impact on resident experience, staff well-being, and equity remains limited and mixed, indicating that technology alone cannot resolve the structural challenges facing long-term care. For practitioners and administrators, AI should therefore be adopted as a tool to augment—not replace—clinical judgment, frontline staffing, and person-centered care practices.

For researchers, the findings highlight several priorities. Future studies should move beyond proof-of-concept designs and conduct head-to-head comparisons of AI-enabled and non-AI models of care using standardized, multidimensional quality metrics. Multi-site and longitudinal designs that include diverse resident populations are needed to clarify generalizability, equity implications, and long-term effects on outcomes such as functional status, quality of life, and staff retention. In addition, implementation research that explicitly examines workflow integration, training strategies, and organizational culture will be critical to understanding why similar AI tools succeed in some facilities but not others.

Policymakers and regulators can support high-quality AI adoption in nursing homes by establishing clear governance frameworks for data use, transparency, and accountability, while also investing in digital infrastructure for under-resourced facilities. Engaging residents, family members, and direct-care workers as partners in the design, selection, and evaluation of AI systems will help ensure that innovations reflect the values and preferences of those most affected. Collectively, these steps can help translate the promising but uneven evidence reviewed here into practical, trustworthy AI applications that advance high-quality, person-centered care in nursing homes.

## 6. Study Limitations

This review is subject to several limitations. The heterogeneity of study designs, settings, and outcome measures across included articles limited the feasibility of meta-analytic synthesis, and findings should be interpreted with appropriate caution regarding generalizability. Several included studies were conducted within single facilities or specific national contexts, limiting the external validity of the reported outcomes. Future research should prioritize multi-site, longitudinal studies with diverse resident populations to more robustly evaluate the real-world impact of AI on nursing home quality of care.

Reviewer subjectivity: Although at least three independent researchers reviewed each article for inclusion, and consensus was required for all data extraction decisions, the thematic coding process remains subject to interpretive subjectivity. No formal inter-rater reliability coefficient (e.g., Cohen’s kappa) was calculated prior to consensus resolution, which limits the reproducibility of the thematic categorization. Future reviews in this area should prospectively pilot the coding framework and report inter-rater reliability statistics before full data extraction.

Database and search limitations: The search was conducted across four databases—PubMed, CINAHL Ultimate, Academic Search Complete, and Nursing and Allied Health Reference Source. Notably, Nursing and Allied Health Reference Source yielded no unique results, and all articles from Academic Search Complete were duplicates of PubMed results, meaning the effective evidence base was drawn almost entirely from PubMed and CINAHL Ultimate. This concentration increases the risk that relevant articles indexed in other platforms (e.g., Embase, Scopus, Web of Science, IEEE Xplore for technical AI literature) were not captured. The use of a single, uniform Boolean string across databases—while promoting consistency—may have further constrained yield in databases with different indexing vocabularies. Researchers replicating or extending this work should consider database-specific subject headings (e.g., MeSH terms in PubMed) and a broader set of databases to improve comprehensiveness.

## Figures and Tables

**Table 1:** Johns Hopkins Nursing Evidence-Based Practice Model ratings for each article.

Year	Author	Strength	Quality
2022	Zhao et al. [9]	III	B
2025	Beltran et al. [10]	III	B
2020	Lindbergh et al. [15]	II	A
2025	Barrett et al. [16]	II	B
2024	Jain et al. [17]	II	A
2024	Zhao et al. [18]	II	A
2025	Boyle et al. [19]	III	B
2025	Fernandes et al. [20]	III	B
2025	Russell et al. [21]	II	B
2024	Ni et al. [22]	III	B
2024	Rudolph et al. [23]	II	B
2024	Caggiari et al. [24]	II	B
2019	Libertucci et al. [25]	II	B
2025	Patrascu et al. [26]	II	B
2025	Palace et al. [27]	II	B
2020	Lanza et al. [28]	III	B
2024	Dowd et al. [29]	II	A
2025	Milford et al. [30]	III	B
2025	Ase et al. [31]	II	B
2025	Jimenez-Arroyo et al. [32]	II	B
2025	Abdulai et al. [33]	III	B
2025	Lefelle et al. [34]	II	B
2025	Lin et al. [35]	III	B
2021	Zhao et al. [36]	II	A
2023	Shin et al. [37]	II	B
2025	Ronan et al. [38]	III	B
2025	Ronan et al. [39]	III	B

**Table 2:** Mixed Methods Appraisal Tool ratings for each article.

Mixed Methods Appraisal Tool (MMAT) Version 2018—Criterion-by-Criterion Appraisal											
Ref. #	Author, Year	MMAT Category	S1 Clear Research Question?	S2 Data Address the RQ?	Criterion 1	Criterion 2	Criterion 3	Criterion 4	Criterion 5	# Yes (of Applicable Criteria)	Notes/Key Methodological Limitations
[9]	Zhao et al., 2022	*Review/Scoping—MMAT Not Applicable*	*NA*	*NA*	*NA*	*NA*	*NA*	*NA*	*NA*	*N/A*	*Scoping review—MMAT not applicable.*
[10]	Beltran et al., 2025	*Review/Scoping—MMAT Not Applicable*	*NA*	*NA*	*NA*	*NA*	*NA*	*NA*	*NA*	*N/A*	*Scoping review—MMAT not applicable.*
[15]	Lindberg et al., 2020	Quantitative Non-Randomized (Cat. 3)	Y	Y	Y	Y	Y	N	Y	**6/7**	*Single-facility EHR dataset; no blinding; ML model not externally validated.*
[16]	Barrett et al., 2025	Quantitative Non-Randomized (Cat. 3)	Y	Y	Y	Y	Y	CT	Y	**6/7**	*Difference-in-differences design; single health system; partial confounder control.*
[17]	Jain et al., 2024	Quantitative Non-Randomized (Cat. 3)	Y	Y	Y	Y	Y	CT	Y	**6/7**	*Single-facility pilot; small sample; no control group.*
[18]	Zhao et al., 2024	Mixed Methods (Cat. 5)	Y	Y	Y	Y	CT	Y	Y	**6/7**	*Mixed methods; single-country sample (China); self-reported acceptability data.*
[19]	Boyle et al., 2025	*Review/Scoping—MMAT Not Applicable*	*NA*	*NA*	*NA*	*NA*	*NA*	*NA*	*NA*	*N/A*	*Umbrella review—MMAT not applicable.*
[20]	Fernandes et al., 2025	*Review/Scoping—MMAT Not Applicable*	*NA*	*NA*	*NA*	*NA*	*NA*	*NA*	*NA*	*N/A*	*Scoping review—MMAT not applicable.*
[21]	Russell et al., 2025	Quantitative Descriptive (Cat. 4)	Y	Y	Y	CT	Y	CT	*NA*	**4/6**	*Protocol paper; primary outcome data not yet collected; feasibility design only.*
[22]	Ni et al., 2024	Quantitative Descriptive (Cat. 4)	Y	Y	N	Y	Y	*NA*	*NA*	**4/5**	*Single case study; n=1; extremely limited generalizability.*
[23]	Rudolph et al., 2024	Quantitative Non-Randomized (Cat. 3)	Y	Y	Y	Y	Y	CT	Y	**6/7**	*Non-random convenience sample; no blinding of AI* vs. *specialist raters.*
[24]	Caggiari et al., 2024	Quantitative Non-Randomized (Cat. 3)	Y	Y	Y	Y	CT	N	Y	**5/7**	*Observational; confounders not controlled; community-dwelling sample.*
[25]	Libertucci et al., 2019	Quantitative Descriptive (Cat. 4)	Y	Y	CT	Y	Y	*NA*	*NA*	**4/5**	*Pilot study; convenience sample; no control group; limited statistical power.*
[26]	Patrascu et al., 2025	Quantitative Non-Randomized (Cat. 3)	Y	Y	Y	Y	Y	CT	Y	**6/7**	*Secondary data analysis; dementia subgroup only; limited outcome completeness.*
[27]	Palace et al., 2025	Quantitative Descriptive (Cat. 4)	Y	Y	Y	CT	Y	*NA*	*NA*	**4/5**	*Feasibility study; small n; no comparator arm.*
[28]	Lanza et al., 2020	*Review/Scoping—MMAT Not Applicable*	*NA*	*NA*	*NA*	*NA*	*NA*	*NA*	*NA*	*N/A*	*Narrative/conceptual review—MMAT not applicable.*
[29]	Dowd et al., 2024	Mixed Methods (Cat. 5)	Y	Y	Y	Y	Y	Y	Y	**7/7**	*Cross-national (AU/JP) mixed methods; pain management proxies; self-report bias.*
[30]	Milford et al., 2025	Qualitative (Cat. 1)	Y	Y	Y	Y	Y	Y	Y	**7/7**	*Ethics commentary; qualitative analysis of theoretical arguments; no participant data.*
[31]	Ase et al., 2025	Quantitative Descriptive (Cat. 4)	Y	Y	Y	Y	CT	*NA*	*NA*	**4/5**	*Cross-sectional survey; self-reported dietary data; non-response not assessed.*
[32]	Jiménez-Arroyo et al., 2025	*Review/Scoping—MMAT Not Applicable*	*NA*	*NA*	*NA*	*NA*	*NA*	*NA*	*NA*	*N/A*	*Narrative review—MMAT not applicable.*
[33]	Abdulai et al., 2025	Quantitative Descriptive (Cat. 4)	Y	Y	Y	CT	Y	*NA*	*NA*	**4/5**	*Descriptive modeling study; sampling strategy not fully described.*
[34]	Lefelle et al., 2025	Qualitative (Cat. 1)	Y	Y	Y	Y	CT	Y	Y	**6/7**	*Qualitative content analysis of care interactions; small convenience sample.*
[35]	Lin et al., 2025	Qualitative (Cat. 1)	Y	Y	Y	Y	Y	Y	CT	**6/7**	*Qualitative interview study; purposive sample; transferability limited.*
[36]	Zhao et al., 2021 [protocol]	Mixed Methods (Cat. 5)	Y	Y	CT	Y	Y	Y	CT	**5/7**	*Mixed-methods protocol study; data collection not yet complete at publication.*
[37]	Shin and Jung, 2023	Quantitative Descriptive (Cat. 4)	Y	Y	Y	Y	Y	*NA*	*NA*	**5/5**	*Heuristic usability evaluation; expert panel only; no resident/patient input.*
[38]	Ronan et al., 2025	*Review/Scoping—MMAT Not Applicable*	*NA*	*NA*	*NA*	*NA*	*NA*	*NA*	*NA*	*N/A*	*Narrative review—MMAT not applicable.*
[39]	Ronan et al., 2025	*Review/Scoping—MMAT Not Applicable*	*NA*	*NA*	*NA*	*NA*	*NA*	*NA*	*NA*	*N/A*	*Scoping review—MMAT not applicable.*

*Review/scoping articles rated N/A. Ratings: Y = Yes/N = No/CT = Cannot Tell/N/A = Not Applicable (review/scoping article).* Rating key: Y = Yes (criterion met)/N = No (criterion not met)/CT = Cannot Tell (insufficient information)|N/A = Not Applicable (review/scoping article—MMAT designed for empirical studies only).

**Table 3:** Comparative influence of AI applications on quality-of-care domains in nursing homes.

Thematic Domain (AI Role)	Primary Quality-of-Care Domain(s) Affected	Typical Comparator in Included Studies	Direction of Effect of AI vs. Comparator on Quality of Care
Theme 1: Clinical management, risk prediction, monitoring	Safety (falls, deterioration), effectiveness	Traditional risk scales, manual surveillance, retrospective chart review.	Generally favorable: earlier risk identification, fewer major falls, more proactive care planning.
Theme 2: Pressure injury, wound management, diagnostics	Safety (pressure injuries), effectiveness, timeliness	Standard mattresses, in-person wound rounds, radiologist-dependent imaging.	Favorable for pressure-injury prevention and timeliness of diagnosis; accuracy comparable to specialists in some imaging tasks.
Theme 3: Objective assessment, mental health, end-of-life	Effectiveness, patient experience, safety (appropriate psychotropic use)	Usual documentation, standard psychosocial activities, conventional referral triggers.	Mixed but promising: reductions in depressive symptoms and antipsychotic use; variable impact on broader humanistic outcomes.
Theme 4: Nutrition and personalized daily support	Effectiveness, patient experience, equity (targeting at-risk residents)	Manual dietary recalls, generic nutrition protocols, non-AI SNH concepts.	Emerging evidence: improved detection of unhealthy food patterns and malnutrition risk; impact on clinical outcomes not yet well quantified.
Theme 5: Operational efficiency and staffing	Efficiency, staff well-being, safety (adequate staffing, workload)	Traditional staffing ratios and scheduling, non-AI communication workflows.	Favorable for efficiency and task redistribution; inconsistent effects on staff burden and satisfaction.
Theme 6: Technical, infrastructure, economic considerations	Equity, safety, reliability	Facilities without advanced IoT/AI infrastructure	Neutral to negative: resource and infrastructure gaps may widen quality disparities when AI is unevenly deployed.
Theme 7: Social, ethical, demographic considerations	Equity, patient experience, trust	Non-AI care models sensitive to cultural and demographic differences.	Cautionary: risks of bias, low acceptability, and misalignment with resident preferences if AI is not co-designed.

## Data Availability

The data presented in this study are available in the articles listed in the references.
